# Precision Diagnosis of Viral Respiratory Infections: Unleashing the Power of Multiplex Polymerase Chain Reaction (PCR) for Enhanced Infection Management

**DOI:** 10.7759/cureus.73100

**Published:** 2024-11-05

**Authors:** Minakshi Singh, Arushi Gupta, Aditi Agarwal, Vanya Singh, Balram Omar

**Affiliations:** 1 Microbiology, All India Institute of Medical Sciences, Rishikesh, Rishikesh, IND

**Keywords:** biofire filmarray, multiplex pcr, point-of-care test, severe acute respiratory syndrome coronavirus-2 (sars-cov-2), upper respiratory tract infections, viral respiratory tract infections

## Abstract

Background: Viruses are key agents causing respiratory illnesses, creating significant public health challenges worldwide. Timely identification of the responsible pathogen is crucial for effective patient management. Conventional virus detection methods in laboratories are often laborious and time-consuming, prompting the need for more efficient diagnostic techniques. The multiplex polymerase chain reaction (PCR) method, capable of testing multiple pathogens simultaneously, offers a promising solution.

Objective: This study investigates the efficacy of multiplex PCR, specifically using the BioFire FilmArray Respiratory Panel (RP2.1), in diagnosing viral respiratory infections and its impact on patient outcomes.

Methods: A retrospective study was conducted at the Department of Microbiology, All India Institute of Medical Sciences, Rishikesh, from January 2022 to December 2023, involving 36 patients with upper respiratory tract infection symptoms. Nasopharyngeal and oropharyngeal swabs were collected and tested using the BioFire FilmArray RP2.1, targeting 17 viruses and four bacteria.

Results: Among the 36 samples, 21 (58.33%) exhibited viral agents, with human rhinovirus/enterovirus being the most prevalent (19.44%). Respiratory syncytial virus (RSV), severe acute respiratory syndrome coronavirus-2 (SARS-CoV-2), and influenza B were commonly identified. The study found a single viral etiology in 17 samples (47.22%), dual infections in two samples (5.56%), and triple infections in two samples (5.56%). Patient outcomes varied, with seven recorded deaths, mainly among ICU patients. SARS-CoV-2 was a prominent cause of mortality. Most patients with viral respiratory illnesses were from the pediatric (27.78%) and elderly (19.44%) age groups.

Conclusion: The BioFire FilmArray RP2.1 is a valuable tool for rapid and accurate diagnosis of respiratory infections, facilitating timely and appropriate medical interventions. Despite its higher cost, its efficiency in diagnosis and potential to reduce unnecessary treatments highlight its cost-effectiveness in clinical settings.

## Introduction

Viruses play a pivotal role as primary contributors to respiratory illnesses, exerting a global impact and presenting substantial challenges in the realm of public health [1]. These viruses, belonging to various families, possess a remarkable ability to spread easily from person to person and are found globally [2]. Among the widely circulating respiratory viruses are influenza virus, respiratory syncytial virus (RSV), parainfluenza viruses, metapneumovirus, rhinovirus, coronaviruses, adenoviruses, and bocaviruses, causing regular infections or outbreaks on a global scale [3].

Clinical manifestations of respiratory tract infections result from a diverse array of pathogens, including bacteria and viruses, with associated symptoms often lacking specificity for a particular pathogen. Virus detection through conventional methods has proven challenging in laboratories, necessitating expertise and time [4]. In clinical settings, identifying the etiological agent promptly is crucial for precise and timely patient management. Molecular methods, such as polymerase chain reaction (PCR) and sequencing, expedite the detection of etiological agents. However, considering the potential involvement of various pathogens, conducting investigations through specific PCR assays for individual viruses ("monoplex PCR") becomes impractical due to its time-consuming and intricate nature in the laboratory.

The rising significance of multiplex PCR methods lies in their ability to test for numerous pathogens concurrently in a single analysis. This approach is increasingly favored as it offers comparable specimen volume, time efficiency, and cost-effectiveness to monoplex PCR despite simultaneously analyzing numerous pathogens. In this context, this study delves into the application of multiplex PCR to transform viral respiratory infection diagnostics and enhance patient management.

## Materials and methods

This retrospective study was performed in the Department of Microbiology at All India Institute of Medical Sciences, Rishikesh, India, from January 2022 to December 2023. Data were gathered using the BioFire FilmArray Respiratory Panel (RP 2.1) (BioFire Diagnostics, Salt Lake City, USA) based on requisitions received in the lab along with consent over a period of one year. The analysis encompassed 36 patients showing symptoms of respiratory tract infections from various inpatient departments (IPDs), high-dependency units (HDU), and intensive care units (ICUs). Demographic information and clinical histories of the patients were documented. Nasopharyngeal (NP) and oropharyngeal (throat) swabs were collected in viral transport media (VTM) and promptly processed in the laboratory for multiplex PCR testing.

The BioFire FilmArray Respiratory Panel (RP 2.1), designed to target 21 prevalent causative agents comprising 17 viruses and four bacteria was used. The targeted viruses included adenovirus, coronavirus 229E, coronavirus HKU1, coronavirus NL63, coronavirus OC43, severe acute respiratory syndrome coronavirus 2 (SARS-CoV-2), human metapneumovirus, human rhinovirus/enterovirus, influenza A (including subtypes H1, H1-2009, and H3), influenza B, parainfluenza virus 1, parainfluenza virus 2, parainfluenza virus 3, parainfluenza virus 4, and RSV. Bacterial targets comprised *Bordetella parapertussis* (IS1001), *Bordetella pertussis (ptxP), Chlamydia pneumoniae, and Mycoplasma pneumoniae* [5].

Appropriate personal protective equipment (PPE) was used while handling Biofire RP panel pouches and samples. The pouch was opened and hydrated per the manufacturer’s instructions [5]. Briefly, the VTMs were mixed thoroughly by inversion. 0.3 mL of the specimen from the VTM was added to the sample buffer in the sample injection vial. The sample mix was loaded, and the pouch was run. There were two inbuilt pouch controls: RNA Process Control and PCR2 Control. The BioFire RP2.1 test report was automatically displayed upon completion of the run. A descriptive analysis was done. If specific numbers or detailed breakdowns were available in referenced studies, we could adjust per their reported frequencies over time. Without specific samples from the mentioned studies, we use our known 36 patients for empirical analysis directly. This is a limitation of our study.

Inclusion criteria included all patients for whom a BioFire FilmArray Respiratory Panel (RP2.1) requisition was received in the lab.

Exclusion criteria included patients unwilling to participate in the study and instances where controls failed in the BioFire FilmArray Respiratory Panel (RP2.1).

## Results

The demographic details of the patients are represented in Table 1. Maximum patients were from the pediatric age group of 0-10 years (27.78%), followed by the elderly age group of >61 years (19.44%). The lowest number of patients was from the age group of 31-40 years (5.56%). Females (55.56%) predominated over males (44.44%) in this study. Among the 36 patients, 17 patients (47.22%) were admitted to various IPDs, 17 patients (47.22%) to multiple ICUs, and two patients (5.56%) were admitted to HDU (Table 2). All the patients complained of respiratory tract infections, including one or more of fever, cough, shortness of breath, chest pain, orthopnea, headache, and nasal discharge (Table 3).

**Table 1:** Demographic details of the patients recruited in the study (N=36)

Age group (in years)	Number of patients (36)	Males (16)	Females (20)
0-10	10 (27.78%)	6	4
11-20	5 (13.89%)	4	1
21-30	5 (13.89%)	2	3
31-40	2 (5.56%)	1	1
41-50	3 (8.33%)	0	3
51-60	4 (11.11%)	1	3
>61	7 (19.44%)	2	5

**Table 2:** Distribution of patients by various clinical departments (N=36)

Department	Type	Number	Total
Intensive care unit (ICU)	Pulmonary ICU	8 (22.22%)	17 (47.22%)
	Pediatric ICU	6 (16.66%)	
	Medicine ICU	1 (2.7%)	
	Neonatal ICU	1 (2.7%)	
	Critical care unit	1 (2.7%)	
High dependency unit (HDU)		2 (5.56%)	2 (5.56%)
Various in-patient departments (IPDs)	Pediatric medicine ward	4 (11.11%)	17 (47.22%)
	Medical oncology	4 (11.11%)	
	Geriatric medicine ward	3 (8.33%)	
	Pulmonary medicine ward	2 (5.56%)	
	Nephrology ward	2 (5.56%)	
	General medicine ward	2 (5.56%)	
Total		36 (100%)	36 (100%)

**Table 3:** Distribution of patients according to signs and symptoms clinically (N=36)

Symptoms/signs	Number of patients
Fever	33 (91.6%)
Shortness of breath	32 (88.8%)
Cough	30 (83.33%)
Chest pain	3 (8.83%)
Headache	3 (8.83%)
Orthopnea	2 (5.56%)
Pedal oedema	2 (5.56%)
Palpitations	1 (2.78%)

The results of multiplex PCR are represented in Tables 4-5. Among the 36 samples tested, 21 (58.33%) samples exhibited the presence of viral agents. In seven (19.44%) samples, human rhinovirus/enterovirus was identified, followed by RSV in five (13.89%) samples, SARS-CoV-2 in four (11.11%) samples, influenza B in three (8.33%) samples, influenza A H1-2009 in two (5.56%) samples, parainfluenza4 in two (5.56%) samples, influenza A in two (5.56%) samples, and coronavirus 0c43 in one (2.78%) sample.

**Table 4:** Results of multiplex PCR utilizing the BioFire FilmArray Respiratory Panel (RPRv 2.1) (N=36) PCR: polymerase chain reaction

Single-agent results	Number of patients
Human rhinovirus/enterovirus	7 (19.44%)
Influenza A	2 (5.56%)
Influenza B	3 (8.33%)
Influenza A H1-2009	2 (5.56%)
Respiratory syncytial virus (RSV)	5 (13.89%)
Severe acute respiratory syndrome coronavirus-2 (SARS-CoV-2)	4 (11.11%)
Adenovirus	1 (2.78%)
Parainfluenza 4	2 (5.56%)
Coronavirus 0c43	1 (2.78%)
Bacterial agent (*Bordetella parapertussis*)	1 (2.78%)

**Table 5:** Results of multiplex PCR utilizing the BioFire FilmArray Respiratory Panel (RPRv 2.1) depicting single or multiple etiologies (N=36)

Results	Number of patients
Single viral agent	17 (47.22)
Dual viral coinfection	2 (5.56%)
Triple viral coinfection	2 (5.56%)
Bacterial agent	1 (2.78%)
None detected	14 (38.89%)
Total	36 (100%)

A single viral cause was identified in 17 samples (47.22). Additionally, two (5.56%) samples displayed concurrent infection involving two viruses, and another two (5.56%) samples exhibited coinfection with three viruses. *Bordetella parapertussis* was identified as the bacterial etiology in a single sample (2.78%). None of the targeted pathogens were detected in 14 (38.89%) samples (Table 5).

Patients with a single viral etiological agent had the following outcomes: four tested positive for RSV, three for SARS-CoV-2, three for influenza B, one for adenovirus, one for parainfluenza 4, one for influenza A H1-2009, and one for influenza A. Among the 17 patients with a single viral cause, there were 12 females and five males. Dual viral infections were observed in two patients, with one having coinfection with human rhinovirus/enterovirus and RSV, while the other had coinfection with human rhinovirus/enterovirus and influenza A H1-2009. Both cases of dual viral infections were in males. Additionally, two patients exhibited infection with three viral agents, with one presenting a coinfection of coronavirus 0c43, human rhinovirus/enterovirus, and parainfluenza 4. The other patient with triple viral infection was diagnosed with SARS-CoV-2, human rhinovirus/enterovirus, and influenza A coinfection. Both patients with triple viral coinfection were males.

The duration of hospitalization is represented in Table 6. The patient with influenza B experienced the longest hospital stay, lasting 40 days, followed by 37 days for a patient with a triple co-infection of coronavirus 0C43, human rhinovirus/enterovirus, and parainfluenza 4. Conversely, the shortest hospital stays were observed in patients with RSV infection, lasting only one day, followed by three days for patients with influenza A H1-2009 infection.

**Table 6:** Length of hospital stay (in days) in patients suffering from viral respiratory tract infections RSV: respiratory syncytial virus; SARS-CoV-2: Severe acute respiratory syndrome coronavirus-2

Associated virus/es	Number of cases	Length of hospital stay
Adenovirus	1	13 days
Coronavirus 0C43, human rhino/enterovirus, parainfluenza 4	1	37 days
Human entero/rhinovirus	3	15-30 days
Human entero/rhinovirus, RSV	1	31 days
Human rhino/enterovirus, influenza A H1-2009	1	19 days
Influenza A	1	18 days
Influenza B	3	14-40 days
Influenza A H1-2009	1	3 days
Parainfluenza 4	1	21 days
RSV	4	1-18 days
SARS-CoV-2	3	5-28 days
SARS-CoV-2, human rhinovirus, influenza A	1	15 days

Total leukocyte count (TLC) was below normal in two patients, within the normal range in 13 patients, and slightly elevated in six patients. Procalcitonin levels were slightly increased in seven patients, with the highest value being 2.67, while they remained within the normal range in 19 patients with viral respiratory etiologies. Superimposed bacterial infection was observed in six patients with a single viral etiology, identified through aerobic culture.

The clinical outcome of the patients was documented in terms of discharge or mortality. Seven patients succumbed to the illness, while 14 patients were successfully discharged in a hemodynamically stable condition. In patients with a singular viral cause of respiratory illness, there were two fatalities attributed to SARS-CoV-2 infection. Additionally, one fatality each was recorded for patients with RSV, influenza B, parainfluenza 4, RSV, and influenza A H1-2009 infections. Among patients with coinfections, only one death was reported in a patient with triple viral coinfection involving SARS-CoV-2, human rhinovirus, and influenza A (Table 7).

**Table 7:** Mortality associated with viral etiological agents (N=36) RSV: respiratory syncytial virus; SARS-CoV-2: Severe acute respiratory syndrome coronavirus-2

Results	Number of patients
Influenza B	1 (2.78%)
Influenza A H1-2009	1 (2.78%)
Parainfluenza 4	1 (2.78%)
RSV	1 (2.78%)
SARS-CoV-2	2 (5.56%)
SARS-CoV-2, human rhinovirus, influenza A	1 (2.78%)
TOTAL	36 (100%)

Out of the seven reported deaths, five occurred among patients in the ICU, while the remaining two were in IPDs. Both fatalities in the IPD patients were specifically linked to the geriatric ward, where the individuals were notably immunocompromised.

## Discussion

In our study, the largest proportion of patients was in the pediatric age group of 1-10 years (27.78%), followed by individuals aged over 61 years (19.44%). Other age categories comprised 11-20 years (13.89%), 21-30 years (13.89%), 51-60 years (11.11%), 41-50 years (8.33%), and 31-40 years (5.56%). Females constituted 55.56% of the cases, exceeding the male percentage of 44.44% (refer to Table 1). This data aligns with a study conducted in the USA by Leber et al., where the majority of specimens were from pediatric subjects: 55% were from children aged five years and under, 21% from those aged 6 to 21 years, 17% from adults over 50 years, and 8% from adults aged 22 to 49 years [6]. This underscores the prevalence of suspected viral respiratory infections among pediatric and elderly individuals who may be immunocompromised.

Overall, the predominant viral agent was human rhinovirus/enterovirus (19.44%), followed by RSV (13.89%), SARS-CoV-2 (11.11%), influenza B (8.33%), influenza A H1-2009 (5.56%), parainfluenza 4 (5.56%), influenza A (5.56%), and coronavirus 0c43 (2.78%). These results are consistent with the findings reported by Appak et al. from Turkey in 2019, Leber et al. from the USA, and Leli et al. from Italy in 2021, where human enterovirus/rhinovirus emerged as the predominant agent, followed by RSV [6-8].

A single viral cause was identified in 17 samples (47.22%). Leli et al. from Italy reported monomicrobial infection in 80.4% of patients [8]. Two (5.56%) samples displayed coinfection with two viruses, and another two (5.56%) samples exhibited concurrent infection involving three viruses. No significant differences were found in the detection rate between males and females. Otherwise, according to age range, samples collected from the pediatric group were more frequently positive compared to adults and elderly, respectively. These findings are consistent with the findings of Leli et al. from Italy [8].

The patient outcomes and lengths of hospital stay varied across different viral infections. Among the cases reviewed, those infected with SARS-CoV-2 experienced diverse outcomes, with one patient succumbing to the infection after five days of hospitalization, while another endured a 28-day stay but eventually passed away. Parainfluenza 4 claimed the life of a 15-year-old patient after 21 days in the hospital. Influenza A H1-2009 resulted in a relatively shorter hospital stay of three days before proving fatal for a 66-year-old individual. Conversely, patients with RSV infection had shorter hospital stays, with one discharged after eight days and another after just three days. Similarly, cases of influenza B led to varying outcomes, with one patient surviving a 14-day hospitalization while another endured a 40-day stay but ultimately perished. Other viral infections, such as coronavirus 0C43, human rhino/enterovirus, and parainfluenza 4, resulted in a 37-day hospital stay, but the patient was discharged. Infants and toddlers infected with SARS-CoV-2 or human enterovirus/rhinovirus comparatively had shorter stays, with one five-month-old discharged after 15 days of hospitalization with infection of human enterovirus/rhinovirus and a three-month-old discharged after nine days of hospitalization with infection of SARS-CoV-2. In contrast, an adult patient infected with human rhino/enterovirus was discharged after a 31-day hospitalization. These cases illustrate the variability in outcomes and lengths of hospital stays associated with different viral infections, influenced by factors such as age, co-morbidities, and viral strain.

A total of seven deaths were reported among patients diagnosed with viral respiratory tract infections. Among them, the SARS-CoV-2 infection was prominent, contributing to the deaths of five individuals across different age groups, including a 37-year-old female, a 15-year-old male, a 66-year-old female, a 73-year-old female, and an infant aged six months. Additionally, Influenza A H1-2009 infection led to fatal outcomes in a 66-year-old female, while parainfluenza 4 infection resulted in the death of a 15-year-old male. These cases underscore the severity and diverse impact of viral respiratory infections on patient outcomes, emphasizing the importance of timely and effective medical interventions.

Out of the 21 patients diagnosed with viral respiratory illness, five were not initiated on antiviral therapy, while the remaining 16 received such treatment. The most commonly prescribed antiviral medications were ribavirin and oseltamivir. Among those who received antiviral therapy, three deaths were recorded. Two of these fatalities were attributed to SARS-CoV-2, occurring in patients from the pediatric ICU and medicine ICU, while one was linked to RSV in an elderly debilitated patient.

Four deaths were reported among patients who did not receive antiviral treatment. Three of these deaths were associated with a single viral cause of respiratory tract infection, specifically, influenza A H1 2009, influenza B, and parainfluenza 4. Additionally, one death was reported in a pediatric patient who had a triple coinfection involving SARS-CoV-2, influenza A, and human rhino/enterovirus.

Due to the limited existing literature on the topic, we were unable to compare the scenario of our setup with other regions in India. Additionally, this study was carried out at a single center, unlike multicenter studies, which restricted our ability to generalize the efficacy of the findings. Moreover, the lack of availability of monoplex PCR and viral cultures hindered our assessment of the diagnostic accuracy of the multiplex PCR utilized.

As the BioFire FilmArray Respiratory Panel (RP 2.1) is a multiplex PCR, it is therefore a very expensive test. This test is highly specific to the point-of-care test (POCT), which is not affordable by a larger part of the population. This test is only available in select centers of India, including our tertiary care institute. This is a limitation of our study.

## Conclusions

This study highlights the significant role of multiplex PCR in the accurate and efficient diagnosis of viral respiratory infections. The findings underscore the prevalence of respiratory viruses, particularly human rhinovirus/enterovirus and RSV, among pediatric and elderly populations, emphasizing the need for targeted interventions in these vulnerable groups. The ability to simultaneously detect multiple pathogens not only facilitates timely diagnosis but also enhances patient management strategies, potentially improving clinical outcomes. Our data reveal a concerning mortality rate associated with specific viral infections, particularly SARS-CoV-2, influenza A, and parainfluenza 4. The variability in hospitalization duration and outcomes further illustrates the complexities of managing viral respiratory illnesses. Importantly, the use of antiviral therapies showed mixed results, suggesting the necessity for ongoing research to optimize treatment protocols.

Overall, the adoption of multiplex PCR technologies in clinical settings represents a transformative approach to managing viral respiratory infections, ultimately contributing to improved public health responses and patient care. Future studies should focus on longitudinal assessments of viral infections and the effectiveness of therapeutic interventions to enhance our understanding and management of these pervasive respiratory diseases.
